# Unconditional government cash transfers in support of orphaned and vulnerable adolescents in western Kenya: Is there an association with psychological wellbeing?

**DOI:** 10.1371/journal.pone.0178076

**Published:** 2017-05-31

**Authors:** Sylvia Shangani, Don Operario, Becky Genberg, Kipruto Kirwa, Miriam Midoun, Lukoye Atwoli, David Ayuku, Omar Galárraga, Paula Braitstein

**Affiliations:** 1 Brown University, Department of Behavioral Social Sciences, Providence, Rhode Island, United States of America; 2 Moi Teaching and Referral Hospital, Eldoret, Kenya; 3 Academic Model Providing Access to Healthcare (AMPATH), Eldoret, Kenya; 4 Brown University, Department of Health Services, Policy and Practice, Providence, Rhode Island, United States of America; 5 Brown University, Department of Epidemiology, Providence, Rhode Island, United States of America; 6 Moi University, College of Health Sciences, School of Public Health, Department of Epidemiology and Biostatistics, Eldoret, Kenya; 7 Moi University, College of Health Sciences, School of Medicine, Department of Behavioral Sciences, Eldoret, Kenya; 8 Moi University, College of Health Sciences, School of Medicine, Department of Medicine, Eldoret, Kenya; 9 University of Toronto, Dalla Lana School of Public Health, Division of Epidemiology, Toronto, Canada; University of Texas Medical Branch at Galveston, UNITED STATES

## Abstract

**Background:**

Orphaned and vulnerable adolescents (OVA) in sub-Saharan Africa are at greater risk for adverse psychological outcomes compared with their non-OVA counterparts. Social interventions that provide cash transfers (CTs) have been shown to improve health outcomes among young people, but little is known about their impact on the psychological wellbeing of OVA.

**Objective:**

Among OVA in western Kenya, we assessed the association between living in a household that received monthly unconditional government CTs and psychological wellbeing.

**Methods:**

We examined the likelihood of depression, anxiety, post-traumatic stress symptoms (PTSS) and positive future outlook among 655 OVA aged between 10 and 18 years who lived in 300 randomly selected households in western Kenya that either received or did not receive unconditional monthly CTs.

**Results:**

The mean age was 14.0 (SD 2.4) years and 329 (50.2%) of the participants were female while 190 (29.0%) were double orphans whose biological parents were both deceased. After adjusting for socio-demographic, caregiver, and household characteristics and accounting for potential effects of participant clustering by sub-location of residence, OVA living in CT households were more likely to have a positive future outlook (odds ratio [OR] 1.47, 95% confidence interval [CI] 1.08, 1.99), less likely to be anxious (OR 0.57, 95% CI 0.42, 0.78), and less likely to have symptoms of post-traumatic stress (OR 0.50, 95% CI 0.29, 0.89). We did not find statistically significant differences in odds of depression by CT group.

**Conclusion:**

OVA in CT households reported better psychological wellbeing compared to those in households not receiving CTs. CT interventions may be effective for improving psychological wellbeing among vulnerable adolescents in socioeconomically deprived households.

## Introduction

Sub-Saharan Africa (SSA) is home to approximately 53.6 million orphaned children, a majority of whom live in extreme poverty [1], often with relatives or guardians of limited means, and in households with many other dependent children [2, 3]. Grandparents are commonly their primary caretakers [4]. Children who live in poor households often do not have access to basic necessities such as shelter, food, clean water, health care, and education [2, 5–7]). In Kenya, there are over 20 million children living in impoverished families that survive on less than 1 US dollar per day. To achieve the goals of the United Nations Children’s Fund (UNICEF) related to children’s rights and development, and the targets of the United Nation’s Sustainable Development Goal 3 to ensure healthy lives and promote well-being for all people at all ages, appropriate socioeconomic policies, services and interventions are required to support poor and vulnerable children in Kenya and across Africa.

Poverty constitutes an important risk factor for adverse mental health outcomes among children, and those who live in the poorest households are more likely to experience negative psychological outcomes [8–10]. Poverty and mental health are inextricably linked (Evans & Cassells, 2014), and poverty in earlier years of life particularly affects cognitive and psychosocial development [11]. To address poverty and its adverse child health and developmental consequences, economic incentives are increasingly used in African countries. A number of studies have shown that cash transfers (CTs), which involve the provision of cash assistance with the objective of increasing a household’s real income [12], can reduce poverty and improve health outcomes of orphaned children [13–15]. CT studies in African countries suggest that economic incentives play an important role in influencing adolescents' sexual risk-taking intentions [16], and that savings-related interventions can improve self-rated health of orphaned adolescents in impoverished settings [17]. Self-rated health is an important correlate of actual future health [18]. Recent data from Malawi demonstrated that unconditional CTs to poor households improved health outcomes for vulnerable children aged 6–17 years [13]. In Kenya, a reduction in transactional sex and increased school enrolment were observed among women who received CTs [14]. These studies support the hypothesis that economic incentives such as CTs may be effective for improving the health outcomes of orphaned and vulnerable children.

The Kenyan government has expanded unconditional CTs to households caring for orphaned and vulnerable children, increasing the number of such households receiving cash assistance from 7,500 in 2007 to 85,891 by December 2010. As of January 2014, the number of households receiving CTs had reached 170, 000 [19]. The program benefits more than 1,800 households in Uasin Gishu county in western Kenya, where the CT program began in 2007. Enrolled households receive a cash payment of 1,500 Kenya shillings (approximately $20 USD) per month, which is equivalent to about 20% of average monthly household expenditure in the region. Given the rapid expansions in the program, it is critical to evaluate the impact of unconditional government CTs on health and wellbeing in this geographic and epidemiologic context.

Although there is evidence that providing financial help may be an effective way to support orphaned children in resource-limited settings [13, 15, 20, 21], few studies have examined the relationship between direct financial support and psychological wellbeing among OVA [22]. Indeed, researchers have recently made appeals for more data that address the complexity of psychosocial wellbeing among OVA across a wide range of outcome indicators [23, 24]. It is important to understand the potential impact of financial incentives on the psychological states of OVA because mental health conditions are among the leading causes of mortality among adolescents living in poverty [25, 26]. In SSA, orphaned adolescents experience a greater risk for adverse psychological outcomes compared to their non-orphaned counterparts [27]. Recent qualitative evidence suggests that CT programs in SSA may have a positive impact on psychosocial well-being, including improvements in educational performance, participation in social life, and empowerment for decision making [22]. However, only one study has examined the effect of CT programs on self-reported depression among young people aged 15–24 years in Kenya, finding a reduction in depression among those in CT households [9]. The effect of CT was significant only for males, especially those between 20–24 years old and those who were orphans. Effects on other key mental health outcomes such as anxiety, post-traumatic stress symptoms (PTSS), and future outlook were not reported. Furthermore, the study only included data from a part of western Kenya that has higher rates of poverty and HIV relative to the rest of the region [28, 29].

Accordingly, we aimed to investigate the association between unconditional CTs and depression as well as three other mental health outcomes, namely PTSS, anxiety, and future outlook among younger adolescents (10–18 years old) in a different part of western Kenya. We hypothesized that OVA living in CT households would perform better across the four measures of psychological wellbeing compared to their counterparts in non-CT households.

## Methods

### OSCAR’s health and wellbeing study

This paper uses baseline data from the Orphan and Separated Children’s Assessments Related to their Health and Well-Being (OSCAR) study. OSCAR is a longitudinal cohort study evaluating the effects of different domestic care environments on the physical and mental health of orphaned and separate children aged 18 years or less at enrolment. The details of OSCAR are provided elsewhere [30], and a brief description is given here. Participant recruitment was initiated in July 2010, and the study is on-going. The study follows a cohort of orphaned and separated children from communities within 8 administrative locations from Uasin Gishu (UG) county of western Kenya, and includes 300 households, 19 Charitable Children’s Institutions (i.e. orphanages), and 100 street-involved children and youth.

### Study setting

UG county is a largely agricultural region located about 375 kilometres northwest of Nairobi. As of 2015, there were approximately 202,291 households in the county, with about 1,044,675 residents, of whom 41.5% were aged 14 years or younger. The majority of the population (61%) lives in rural settings, and approximately 51% live below the Kenyan poverty line (less than 1 US dollar per day).

### Study population, eligibility, sampling and recruitment

The present analysis was restricted to data collected between 2010 and 2013. Participants were aged 10 to 18 years old, able to communicate in English or Kiswahili (languages used during assessment), and resided in one of the 300 OSCAR households. Children with severe mental or physical health conditions were excluded. Households were recruited using stratified random sampling preceded by extensive community consultations with the aid of community health workers, local area chiefs, and village elders [30]. To obtain a representative sample of households caring for orphans in UG county, we sampled 100 households from each of (i) CT households, (ii) non-CT households from the same sub-location, and (iii) non-CT households from a different sub-location. Sub-locations are administrative regions within counties and that are headed by assistant chiefs. The CT program targets sub-locations that are most socioeconomically deprived. We weighted household numbers to reflect the number of CT or non-CT households caring for orphaned children in each sub-location. To be eligible for the CT program, a household must have at least one orphaned and vulnerable child under 17 years of age and satisfy a set of poverty-related criteria, by exhibiting at least 8 of 13 characteristics related to welfare. These characteristics include the main material of dwelling walls and floors, access to potable water, type of lighting fuel, and ownership of small assets. Additional details of subject eligibility are discussed elsewhere [31].

#### Random selection of CT and non-CT households

The County Children’s Office oversees the government CT program and provided lists of households receiving the government subsidy in each sub-location. For non-CT households, assistant chiefs and village elders generated lists of all the households in their villages and sub-locations caring for orphaned and/or separated children. The lists contained the names of the head of each household, their national ID number (where available), telephone number (where available), the village and sub-location in which they live, the total number of children in the household, and the number of orphaned children in the household. A total of 2,181 households were identified: 1,370 from the non-CT arm (non-CT households from same sub-locations and non-CT households from a different sub-location), and 811 from the CT arm. These lists formed the sampling frame for the random selection of households. Eligible households were required to be caring for orphaned children but may also have been caring for their own biological children. All children in the households were included in the study to abate any distress associated with selection or non-selection to participate in the study. For this analysis, however, we excluded the non-orphaned and non-separated children living with both biological parents.

### Human subjects protection

The study was approved by the Moi University College of Health Sciences/Moi Teaching and Referral Hospital Institutional Research and Ethics Committee, the Indiana University Institutional Review Board, and the University of Toronto Research Ethics Board. Informed consent was provided by the head of household. Individual written assent was provided by all children. Children and guardians who were unable to sign or write their names provided fingerprints. The study dataset contained no identifying information, and all research data were kept confidential.

### Measures and sources of data

We utilized three levels of data: 1) caregiver- and 2) individual-level data from the participants, recorded in clinical and a psychosocial encounter forms, and 3) household-level data. Analyses focused on children categorized as single orphans, double orphans, or separated. A single orphan was defined as a child whose mother or father was deceased, a double orphan as one whose parents were both deceased and a separated child as one for whom at least one parent is completely absent from the child’s life [32].

#### Caregiver and household level data

Caregiver and household data were collected through a standardized site assessment to ascertain the characteristics of the participant’s care environment. The assessment covered general characteristics of the household, children in residence, shelter characteristics, guardian characteristics (age, sex, and education), food and meals, and household food security. Household shelter type (temporary, semi-permanent, permanent or other) was also recorded. The site assessment was administered in person and *in situ* to heads of households by trained Community Health Workers (CHWs). CHWs are residents of the locations in which they work and have in-depth understanding of the cultural context of their communities. Often, they have developed rapport and trust with many households. The assessments were validated through random household audits.

#### Household food security

We used the Household Food Insecurity Access Scale (HFIAS), specifically adapted by the USAID Food and Nutrition Technical Assistance (FANTA) project for use in Developing Countries [33]. Participants completed a nine-item measure, with questions addressing three domains of experience with food insecurity: anxiety and uncertainty about food supply, insufficient quality and variety of food, and insufficient food intake and its implications. Responses to each question ranged from “never”, “rarely”, “sometimes”, and “often” worried. Scores ranged from 0 to 27, where higher values indicate more serious food insecurity. The Cronbach’s alpha was 0.86, suggesting high reliability. We then dichotomized individuals as being food insecure or food secure following the recommendations of the FANTA guide [33], whereby based on their scores on various items, individuals are classified as food insecure if they worried about insufficient food access, insufficient food quality, cut back on quantity of food consumed, did not have food to eat, or experienced frank hunger.

#### Individual level data

Socio-demographic data were collected by a study social worker while clinical assessments were conducted by clinical staff. Data were documented on a standardized study form, including age, enrolment date, sex, ethnicity, religiosity, orphan status, school attendance, number of siblings in the household, length of stay with the caregiver, number of care givers, hospitalization in the past year, and history of child sexual abuse.

#### Outcome variables

Our outcome was psychological wellbeing, assessed using four variables: depression, anxiety, post-traumatic stress symptoms (PTSS), and future outlook.

Depression: Depression was measured using the shortened 11-item version of the Child Depression Inventory (CDI) [34], which has been validated in studies of young people in SSA [35, 36]. For each question asked, adolescents chose one of three statements that best described their feelings (e.g., “I am sad once in a while”; “I am sad many times”; “I am sad all the time”). Statements indicating no symptoms of depression (e.g., “I am sad once in a while”) were coded as 0, statements representing moderate depressive feelings (e.g., I am sad many times) were coded as 1, and statements representing higher levels of depression (e.g., “I am sad all the time”) were coded as 2. The total score was computed by summing up the score across all the 11 items. The possible range of scores was 0 to 22, with a higher score indicating higher levels of depression.

Anxiety: Anxiety was assessed using the 28-item Children’s Manifest Anxiety Scale–Revised [37]. It consists of 28 questions with ‘Yes/No’ responses. The total anxiety score was determined by the number of ‘Yes’ responses. Possible scores ranged from 0 to 28. This scale has been used in South Africa [36].

PTSS: We measured PTSS using the 28-item Amaya-Jackson’s child PTSD checklist (Amaya-Jackson, 2000), which is based on DSM-IV TR criteria. Each item is scored either 0 (never), 1 (Sometimes), 2 (Often) or 3 (All the time). Possible scores ranged from 0 to 84, with higher scores indicating greater symptom occurrence and severity. This scale has been used with orphan children in western Kenya [38] and in South Africa [39].

Future outlook: Future outlook was assessed using a single item measure which asked participants “How do you feel about your future opportunities to be successful and prosper?” Response options included “Your opportunities are limitless”, “You have many opportunities”, “Your opportunities are very limited”, and “You have no opportunities at all”. This measure has been previously used in the National Survey of South African Youth [40] [41] and specifically in studies of orphaned adolescents in South Africa [36, 42].

### Statistical analysis

We conducted descriptive and correlation analyses to characterize socio-demographic, caregiver, household and psychological variables by CT status. We used chi-square and Fisher’s exact tests for categorical covariates, t-test for continuous normally distributed variables and Mann-Whitney Wilcoxon test for non-normally distributed variables. Our primary analysis used multivariable logistic regression models to examine the association between cash transfer and psychological outcomes, adjusting for socio-demographic, household and caregiver characteristics. We accounted for the potential effects of clustering of study participants in administrative geographic units by including fixed effects on sub-location in all regression models. For depression, anxiety, and PTSS outcomes, we created a binary classification by categorizing participants with scores in the top quintile as having experienced the respective outcome. The Cronbach’s alpha for the three outcomes were 0.70, 0.89, and 0.93, respectively. We dichotomized responses for future outlook as having many or limitless opportunities (positive outlook) versus limited or no opportunities. To assess whether the results were robust to our choices of outcome variable categories, we conducted sensitivity analyses by applying Poisson regression with robust error variances on the continuous or binary (for future outlook) scales of the outcomes. Aside from being applicable to both binary and discrete count outcomes [43], this approach has the advantage of directly yielding risk ratios, which have a more natural interpretation compared to odds ratios. We also accounted for sub-location-level clustering in the sensitivity analyses.

Covariates were entered into the multivariable regression models if they were significant at *p* < 0.20 upon bivariable analysis or if they had been identified in prior studies in similar settings as being related to mental health outcomes of OVAs. The final model included socio-demographic characteristics such as child’s age, gender and orphan status, as well as health status, food security and school attendance variables that were considered as possible modifying factors because of the potential association between health and nutrition and psychological wellbeing [44]. We also included ownership of a pair of shoes because it is one of UNICEF’s indicators of a standard of living conducive to a child’s physical, mental, spiritual, moral and social development [45]. A 2-sided *p* value of 0.05 was considered statistically significant. All analyses were done in Stata 14 (StataCorp LP, College Station, Texas).

## Results

The mean age (standard deviation) of study participants was 14.0 (2.4) years, and 50% of them were female. On average, the number of children in both CT and non-CT households was similar, with 5.4 (1.9) and 5.1 (1.9) children, respectively. Table 1 shows descriptive characteristics and tests of group differences. There were more female participants in CT households (57.6% vs 47.0%). Although majority of participants (71%) were single orphans, orphan status did not differ between the two CT groups. Most participants (89.5%) reported having at least one pair of shoes (92.2% in CT-households compared to 87.9% non-CT households). Children in CT households were more likely to have deceased siblings (14.4% vs 10.4%). Majority of participants (88.1%) viewed religion as important in their life, with no difference in this proportion between the two groups. Additionally, adolescents in CT households were less likely to have engaged in transactional sex or to have been sexually abused (Table 1).

**Table 1:** Demographic, caregiver, household, and psychological characteristics of OVA aged 10–18 by CT status.

Characteristic	Overall	CT	Non-CT	*P* value
	(*N* = 655)	(*n* = 231)	(*n* = 424)	
**Socio-demographic**				
Age (years), mean (SD)	14.0 (2.4)	13.9 (2.3)	14.1 (2.4)	0.36
Gender (female), *n* (%)	329 (50.2)	133 (57.6)	196 (47.0)	0.01
Orphan status, *n* (%)				
Double orphan	190 (29.0)	65 (28.1)	125 (29.5)	0.71
Single orphan	465 (71.0)	166 (71.9)	299 (70.5)	
Enrolled in school (yes), *n* (%)	610 (93.1)	213 (92.2)	397(93.6)	0.49
Number of siblings, mean (SD)	2.0 (0.5)	2.1 (0.5)	2.1 (0.5)	0.34
Deceased siblings (yes), *n* (%)	98 (11.8)	33 (14.4)	44 (10.4)	0.13
Religion important (yes), *n* (%)	577 (88.1)	207 (89.5)	370 (87.3)	0.42
Pair of shoes (yes), *n* (%)	586 (89.5)	212 (92.2)	372 (87.9)	0.09
**Medical**				
Hospitalized, past year (yes), *n* (%)	19 (2.9)	9 (3.9)	10 (2.4)	0.26
Sexually abused (yes), *n* (%)	82 (12.5)	21 (9.2)	61 (14.3)	0.06
Transactional sex (yes), *n* (%)	126 (19.2)	30 (13.0)	96 (22.6)	0.003
**Caregiver**				
Age (years), mean (SD)	49.6 (14.1)	52.4 (13.6)	47.9 (14.1)	0.001
Education level, *n* (%)				
None	141(21.5)	68 (29.5)	73 (17.2)	0.001
Primary	366 (55.9)	134 (58.2)	231 (54.6)	
Secondary and above	148 (22.6)	28 (12.3)	120 (28.3)	
Relationship to caregiver, *n* (%)				
Mother	297 (45.3)	102 (44.3)	195 (45.9)	0.27
Father	43 (6.5)	16 (7.1)	26 (6.1)	
Grandparent	147 (22.4)	55 (23.8)	91 (21.5)	
Other	168 (25.5)	58 (24.9)	112 (26.5)	
Length of stay with caregiver, *n* (%)				
= >5 years	451 (68.8)	169 (73.0)	280 (66.1)	0.11
< 5 years	204 (31.2)	62 (27.0)	144 (33.9)	
**Household characteristics**				
Household size, mean (SD)	5.4 (2.3)	5.3 (2.1)	5.5 (2.5)	0.21
No. of children in household, mean (SD)	5.3 (1.9)	5.4 (1.9)	5.1 (1.9)	0.10
Food secure (yes), *n* (%)	138 (21.2)	64 (27.7)	75 (17.7)	0.003
**Psychological outcomes (dichotomous), *n* (%)**				
Positive future outlook (yes)	365 (67.5)	152 (74.2)	213 (63.4)	0.01
Depression (yes)	324 (56.5)	114 (51.4)	233 (59.4)	0.05
Anxiety (yes)	495 (80.1)	164 (73.9)	331 (83.6)	0.04
PTSS (yes)	451 (72.9)	147 (65.6)	304 (77.0)	0.002
**Psychological outcomes (continuous)**, **mean (SD)**				
Depression score	4.8 (3.8)	4.4 (3)	5 (3)	0.09
Anxiety score	9.3 (6.1)	8.7 (6)	10 (6)	0.07
PTSS score	16.18 (14)	14 (13)	17 (14)	0.05

Abbreviations: CT = cash transfer, PTSS = post-traumatic stress symptoms, OVA = orphaned and vulnerable adolescents, SD = standard deviation

The mean age of caregivers was 49.6 (14.1) years, and CT household caregivers were slightly older than those non-CT household caregivers (52.4 [13.6] vs 47.9 [14.1] years). Approximately half of participants lived with their mothers (45.3%). Most caregivers had no formal education or attained only a primary school level (77.4%), and most OVA (68.8%) had lived for more than five years with their current caregiver. Only about 21.2% of households were considered food secure, including 27.7% of CT households relative to 17.7% of non-CT households. We compared the distribution of several proxies for socioeconomic status between CT and non-CT households and found no statistically significant differences (Table 2).

**Table 2:** Comparison of proxies for socioeconomic status or poverty level among CT and non-CT households contributing participants to the study.

Poverty indicator	Overall	CT	Non-CT	*P* value**
	*N* = 655	*n* = 231	*n* = 424	
Roof (mud), %	12.0	12.7	11.7	0.69
Shelter (temporary-mud/thatch), %	37.4	38.4	36.8	0.68
Unprotected water source*, %	10.1	11.7	9.2	0.31
No electricity, %	14.1	16.5	12.8	0.19

Abbreviations: CT = cash transfer

*Unprotected water source include river, stream, pond, lake, ditch, spring, dam, and water vendor

** *P* value for a test of the differences between CT and non-CT groups

Table 3 shows the results of multivariable logistic regression modelling. Adolescents in CT households had higher odds of positive future outlook (adjusted odds ratio [AOR] 1.47, 95% CI 1.08, 1.99), were less likely to be anxious AOR 0.57 (0.42, 0.78), and less likely to have symptoms of post-traumatic stress AOR 0.50 (0.29, 0.89). Depression was not statistically significantly associated with whether an OVA was living in a CT or non-CT household AOR 0.77 (0.47, 1.27). Higher age was associated with higher odds of PTSS, with an AOR of 1.26 (1.12, 1.42), and females were more likely to report positive future outlook than males AOR 1.62 (1.14, 2.31). OVAs with a history of sexual abuse had higher odds of depression AOR 5.22 (2.65, 10.27) and PTSS (AOR 1.80 [1.07, 3.03]). Sensitivity analysis using Poisson regression with robust error variance yielded similar results, with no material differences (S1 Table).

**Table 3:** Adjusted odds ratios (AOR) and 95% confidence intervals for associations between psychological outcomes and cash transfer among OVA population.

Variable	Positive future outlook (N = 509)	Depression	Anxiety	PTSS
		(N = 578)	(N = 582)	(N = 579)
	AOR (95% CI)	AOR (95% CI)	AOR (95% CI)	AOR (95% CI)
Cash transfer	1.47 (1.08, 1.99)**	0.77 (0.47, 1.27)	0.57 (0.42, 0.78)***	0.50 (0.29, 0.89)**
Non-cash transfer (ref)	1.00	1.00	1.00	1.00
**Socio-demographic**				
Age (years)	0.97 (0.89, 1.07)	0.96 (0.88, 1.04)	1.04 (0.96, 1.13)	1.26 (1.12, 1.42)***
Gender (female)	1.62 (1.14, 2.31)**	0.96 (0.72, 1.29)	0.97 (0.56, 1.71)	0.80 (0.67, 0.95)**
Orphan status				
Double	0.82 (0.45, 1.51)	1.35 (0.78, 2.34)	0.69 (0.37, 1.29)	0.95 (0.51, 1.78)
Single (ref)	1.00	1.00	1.00	1.00
Religion important (yes)	2.06 (1.59, 2.67)***	0.29 (0.16, 0.55)***	0.56 (0.22, 1.43)	0.73 (0.46, 1.14)
Pair of shoes (yes)	0.56 (0.32, 0.98)*	0.64 (0.45, 0.92)**	0.33 (0.10, 1.15)	1.02 (0.49, 2.13)
Enrolled in School	1.45 (0.71, 2.99)	1.04(0.77, 1.38)	1.16 (0.70, 1.93)	2.86 (1.61, 5.09)***
**Medical**				
Hospitalized, past year (yes)	1.78 (0.46, 6.91)	1.50 (0.63, 3.56)	1.93 (0.38, 9.89)	1.91 (0.38, 9.58)
Sexually abused (yes)	0.54 (0.27, 1.06)	5.22 (2.65, 10.27)***	1.80 (1.07, 3.03)*	2.68 (1.21, 5.98)**
Transactional sex (yes)	0.46 (0.15, 1.45)	1.12 (0.67, 1.86)	1.73 (0.74, 4.01)	0.69 (0.40, 1.19)
**Caregiver**				
Age in years	1.01 (0.99, 1.02)	1.01 (0.99, 1.03)	1.00 (0.98, 1.01)	1.00 (0.99, 1.04)
Relationship with caregiver				
Father	0.80 (0.38, 1.67)	0.99 (0.63, 1.56)	0.42 (0.19, 0.92)*	0.64 (0.14, 2.98)
Grandparent	1.51 (1.00, 2.26)*	0.79 (0.58, 1. 10)	1.75 (0.89, 3.43)	0.88 (0.33, 2.33)
Other	0.83 (0.47, 1.49)	1.25 (1.03, 1.51)*	1.51 (0.52, 4.39)	0.97 (0.49, 1.91)
Mother (ref)	1.00	1.00	1.00	1.00
Length of stay with caregiver				
>5 years	0.83 (0.49, 1.40)	1.56 (1.08, 2.26)**	1.01 (0.55, 1.88)	0.99 (0.71, 1.38)
< 5 years	1.00	1.00	1.00	1.00
**Household characteristics**				
Household size	1.01 (0.91, 1.12)	0.92 (0.85, 0.99)*	0.94 (0.86, 1.02)	0.90 (0.84, 0.95)***
Food secure (yes)	1.14 (0.74, 1.76)	0.84 (0.55, 1.28)	2.13 (1.85, 2.46)***	1.35 (0.72, 2.55)

Abbreviations: CI = confidence interval, OR = odds ratio, CT = cash transfer, non-CT = non-cash transfer, PTSS = post-traumatic stress symptoms, OVA = orphaned and vulnerable adolescents

*p<0.05

**p<0.01

***p<0.001

## Discussion

We found that among OVA in western Kenya, living in a household receiving unconditional government CTs was associated with better psychological wellbeing compared to living in a non-CT-receiving household. OVA in CT households were less likely to have anxiety and PTSS, and more likely to report perceptions of positive future outlook compared to their counterparts in households not receiving CTs. Although not attaining statistical significance, we also found that OVAs in CT living households were on average 23% less likely to have depressive symptoms. This finding aligns with the estimates of Kilburn and colleagues who reported that Kenyan youth living in households receiving unconditional cash transfers were 24% less likely to have depressive symptoms compared to those not receiving cash transfers [9]. Few studies have assessed the impact of unconditional government cash transfers on the mental wellbeing of orphaned and vulnerable adolescents in sub-Saharan Africa. A recent review of evaluations of cash transfer programmes concluded that more evidence is needed on the effectiveness of poverty alleviation programs for improvement of mental health [22, 23]. Our study contributes towards filling this gap in knowledge.

These results support our primary hypothesis that in a setting like rural Kenya, CT programs appropriately targeted at poor households may confer psychological benefits to children and adolescents. Our findings are also consistent with previous studies conducted in Uganda which showed that interventions aiming to provide social protection and economic empowerment contributed to improved mental functioning and decreased levels of depression and hopelessness among AIDS-orphaned children [15, 17, 46] Additionally, our findings are also in agreement with those of Baird and colleagues, who found that girls receiving unconditional CT experienced a reduction in psychological distress [47].

This study builds on the growing body of literature on how access to financial resources affects the psychological wellbeing of orphans and vulnerable children, especially in Africa where mental health care infrastructure and services remain highly inadequate. The existing literature indicates that in sub-Saharan Africa, CT programs benefit children’s nutrition, physical growth, help reduce risky sexual behaviors and improve school attendance [8, 9, 13, 14, 17, 20, 48]. Our research contributes to this field by showing that such poverty alleviation programs can play a key role in improving the psychological well-being of OVA. This is especially significant because past evaluations of social cash transfers have generally focused on outcomes related to food security, schooling, sexual risk behaviours, and physical health. Poverty and mental health are mutually linked and increase vulnerability to each other, yet comparatively little attention has been paid to the mental health consequences of poverty. CT programs could be effective in improving psychological outcomes of young and vulnerable people in SSA, where most of these outcomes often go undiagnosed and untreated [26].

Psychological well-being is widely linked to many desirable outcomes, ranging from individual and collective economic productivity, peace, security and general physical health [49]. Our findings may provide a basis for how governmental and non-governmental organizations focus their poverty reduction efforts towards implementing well designed CT programs, including making the improvement of psychological wellbeing one of their core goals. Consequently, governments and non-governmental partners should, whenever possible, collect data on the levels and trends in indicators of psychological wellbeing among OVA in their catchment areas, and monitor whether their interventions affect these outcomes.

By enabling households to meet basic needs, cash transfer program could increase OVA participation in school and other productive community activities, and reduce social isolation and stigma [24]. For instance, adolescents have opportunities to build more interactive and beneficial peer networks [50], which may then improve social wellbeing and general health, potentially strengthening coping strategies against anxiety, depression, and PTSS.

A causal association between participating in an unconditional CT program and reduction in depressive symptoms among young men in Kenya was recently reported [9]. We add to this nascent literature by describing associations between CT and additional indicators of mental health, namely anxiety, PTSS and future outlook. A strength of this study is that the selection of both cash transfer and non-cash transfer households was random, using a well-structured sampling frame [30], therefore reducing potential selection bias. Our sampling scheme enabled us to draw direct comparisons between households caring for OVA that received or did not receive CTs. Secondly, the study comprised adolescents, an important high-risk population that is often understudied because much research focusses on younger or older groups. Evidence-based insight into the health needs and potential preventive and therapeutic interventions for this special population are required. Lastly, we used community health workers to collect household level data, which may help limit some biases associated with participation in the study, such as social desirability bias. Due to their relationships of trust and in-depth knowledge of the local communities, participants are more likely to give them honest responses to our questions. On the other hand, this is a cross-sectional analysis relying on self-reported data, therefore we cannot infer causality. Although all psychological measures had strong internal reliability, the measures were not developed for our specific study population and might have cultural, linguistic, or ecological limitations. In addition, we cannot rule out residual confounding or measurement error, which may have played a role, for example, in the negative association between ownership of a pair of shoes and two of the outcomes of interest. Although results do not change when shoe ownership is excluded from the models, this may be a sign of residual confounding or mismeasured covariates, problems that we are addressing in forthcoming work that includes more detailed quantitative measurement as well as qualitative work.

There is need for further research to understand the emotional and psychological needs of caregivers. It is likely that orphans’ physical, mental, and interpersonal well-being is influenced by their relationships with caregivers, and their community context. Furthermore, additional research to find out whether mental health outcomes differ by gender and age, and whether certain adolescent age brackets are more susceptible to long-term mental health consequences is also warranted. As global economic growth slows down, high poverty rates in the least developed countries persist, and the number of orphaned and vulnerable children and adolescents rises, the need for evidence of the effectiveness of direct financial incentives on physical and mental health outcomes becomes more urgent.

In conclusion, we found that OVA living in households receiving CTs in western Kenya reported better psychological wellbeing relative to their counterparts in households not receiving CT. Our findings complement other studies that point to the positive role of CT programs in promoting health. As suggested by recent finding from Kenya, CTs may be most effective as part of a combination of health promotion strategies for orphaned and vulnerable children [9, 14, 48]. As evidence mounts for a cause and effect relationship between CT programs and mental health outcomes among children in developing countries like Kenya, governmental and non-governmental agencies should be encouraged to expand CT programs to reach a wider pool of eligible households caring for orphaned and vulnerable children, and to make the measurement and evaluation how CT interventions impact these outcomes a central component of their health promotion strategies.

## Supporting information

S1 Table Sensitivity analysis showing adjusted rate ratios (ARR) and 95% confidence intervals for associations between psychological outcomes and cash transfer among OVA population, using Poisson regression with robust error variance. (DOCX)
