# Supplementary material for: Unconditional government cash transfers in support of orphaned and vulnerable adolescents in western Kenya: Is there an association with psychological wellbeing?
Source: PLoS One. 2017 May 31;12(5):e0178076. doi: 10.1371/journal.pone.0178076 (PMC5451046; doi:10.1371/journal.pone.0178076)
Supplement: S1 Table — (DOCX) [file pone.0178076.s001.docx]

**Unconditional Government Cash Transfers in Support of Orphaned and Vulnerable Adolescents in western Kenya: Is There an Association with Psychological Wellbeing?**

Supporting Information

**S1 Table: Sensitivity analysis showing adjusted rate ratios (ARR) and 95% confidence intervals for associations between psychological outcomes and cash transfer among OVA population, using Poisson regression with robust error variance**

| **Variable** | **Positive Future Outlook (N = 513)** | **Depression** | **Anxiety** | **PTSS** |
| --- | --- | --- | --- | --- |
|  |  | **(N = 582)** | **(N = 586)** | **(N = 588)** |
|  | **ARR (95% CI)** | **ARR (95% CI)** | **ARR (95% CI)** | **ARR (95% CI)** |
| Cash transfer | 1.11 (1.04, 1.20)** | 0.94 (0.76, 1.16) | 0.91 (0.83, 0.98)** | 0.88 (0.76, 0.94)** |
| Non-cash transfer (ref) | 1.00 | 1.00 | 1.00 | 1.00 |
| **Socio-demographic** |  |  |  |  |
| Age, in years | 0.99 (0.97, 1.02) | 0.98 (0.95, 0.99)* | 1.02 (1.00, 1.04)* | 1.05 (1.03, 1.07)*** |
| Gender (female) | 1.15 (1.05, 1.26)** | 0.99 (0.89, 1.11) | 1.00 (0.87, 1.14) | 0.93 (0.80, 1.09) |
| Orphan status |  |  |  |  |
| Double | 0.94 (0.80, 1.10) | 1.15 (1.01, 1.31)* | 0.98 (0.93, 1.03) | 0.96 (0.84, 1.11) |
| Single (ref) | 1.00 | 1.00 | 1.00 | 1.00 |
| Religion important (yes) | 1.28 (1.13, 1.46)*** | 0.72 (0.57, 0.91)** | 0.82 (0.70, 0.95)* | 0.91 (0.71, 1.16) |
| Pair of shoes (yes) | 0.86 (0.76, 0.99)* | 0.92 (0.82, 1.02) | 0.84 (0.75, 0.95)** | 0.96 (0.80, 1.16) |
| Enrolled in School | 1.11 (0.87, 1.40) | 1.07 (0.97, 1.19) | 0.92 (0.80, 1.05) | 1.10 (0.85, 1.42) |
| **Medical** |  |  |  |  |
| Hospitalized, past year (yes) | 1.13 (0.92, 1.40) | 1.08 (0.74, 1.58) | 1.16 (0.87, 1.53) | 1.07 (0.82, 1.39) |
| Sexually abused (yes) | 0.78 (0.57, 1.06) | 1.54 (1.30, 1.83)*** | 1.30 (1.16 , 1.45)*** | 1.45 (1.24, 1.68)*** |
| Transactional sex (yes) | 0.75 (0.48, 1.17) | 1.00 (0.89, 1.11) | 1.05 (0.97, 1.13) | 1.09 (0.93, 1.28) |
| **Caregiver** |  |  |  |  |
| Age in years | 1.00 (0.99, 1.01) | 1.01 (1.00, 1.01) | 1.00 (0.99, 1.01) | 1.00 (0.99, 1.00) |
| Relationship with caregiver |  |  |  |  |
| Father | 0.94 (0.77, 1.15) | 0.86 (0.71, 1.04) | 0.96 (0.77, 1.20) | 0.86 (0.52, 1.44) |
| Grandparent | 1.12 (1.01, 1.25)* | 0.92 (0.78, 1. 08) | 1.13 (0.95, 1.35) | 0.93 (0.72, 1.22) |
| Other | 0.95 (0.83, 1.09) | 1.10 (1.02, 1.18)* | 1.16 (0.98, 1.38) | 0.93 (0.76, 1.14) |
| Mother (ref) | 1.00 | 1.00 | 1.00 | 1.00 |
| Length of stay with caregiver |  |  |  |  |
| > 5 years | 0.94 (0.79, 1.12) | 1.13 (0.98, 1.31) | 1.12 (1.01, 1.24)* | 0.91 (0.76, 1.10) |
| < 5 years | 1.00 | 1.00 | 1.00 | 1.00 |
| **Household characteristics** |  |  |  |  |
| Household size | 1.00 (0.97, 1.03) | 0.99 (0.97, 1.02) | 1.01 (0.98, 1.04) | 0.99 (0.96, 1.02) |
| Food secure (yes) | 1.04 (0.92, 1.18) | 1.01 (0.91, 1.12) | 1.15 (1.01, 1.31)* | 1.11 (0.96, 1.28) |

Abbreviations: CI = confidence interval, OR = odds ratio, CT = cash transfer, non-CT = non-cash transfer, PTSS = post-traumatic stress symptoms, OVA = orphaned and vulnerable adolescents

*p<0.05; **p<0.01; ***p<0.001
